# Centrifugation and capillarity integrated into a multiple analyte whole blood analyser

**DOI:** 10.1155/S1463924695000174

**Published:** 1995

**Authors:** C. T. Schembri, T. L. Burd, A. R. Kopf-Sill, L. R. Shea, B. Braynin

**Affiliations:** Abaxis 1320 Chesapeake Terrace Sunnyvale California 94089 USA

## Abstract

A unique clinical chemistry analyser is described which processes 90 μl of whole blood (fingerstick or venous) into multiple aliquots of diluted plasma and reports the results of 12 tests in 14 min. To perform a panel of tests, the operator applies the unmetered sample directly into a single use, 8 cm diameter plastic rotor which contains the required liquid diluent and dry reagents. Using centrifugal and capillary forces, the rotor meters the required amount of blood, separates the red cells, meters the plasma, meters the diluent, mixes the fluids, distributes the fluid to the reaction cuvettes and mixes the reagents and the diluted plasma in the cuvettes. The instrument monitors the reagent reactions simultaneously using nine wavelengths, calculates the results from the absorbance data, and reports the results.


Journal of Automatic Chemistry, Vol. 17, No. 3 (May–June 1995), pp. 99–104

### Centrifugation and capillarity integrated into a multiple analyte whole blood analyser

**C. T. Schembri, T. L. Burd, A. R. Kopf-Sill, L. R. Shea, and B. Braynin**
*Abaxis, 1320 Chesapeake Terrace, Sunnyvale, California 94089, USA*

*A unique clinical chemistry analyser is described which processes 90 µl of whole blood (fingerstick or venous) into multiple aliquots of diluted plasma and reports the results of 12 tests in 14 min. To perform a panel of tests, the operator applies the unmetered sample directly into a single use, 8 cm diameter plastic rotor which contains the required liquid diluent and dry reagents. Using centrifugal and capillary forces, the rotor meters the required amount of blood, separates the red cells, meters the plasma, meters the diluent, mixes the fluids, distributes the fluid to the reaction cuvettes and mixes the reagents and the diluted plasma in the cuvettes. The instrument monitors the reagent reactions simultaneously using nine wavelengths, calculates the results from the absorbance data, and reports the results.*

### Introduction

Rapid availablity of *in vitro* diagnostic results is advantageous to both the physician and the patient. An illness can be diagnosed more quickly and costs due to the illness can be decreased [1]. Turnaround times are shortened, which reduces the likelihood that test results are no longer valid by the time they are received by the physician [2]. Errors inherent in the laboratory testing cycle are also minimized by performing tests near the patient. Eliminating the need to transport samples to a central laboratory reduces such problems as misplaced samples, inaccurate labeling and transcription, improper icing and bagging, and sample degradation [1, 3, 4].

Test systems must produce reliable results, independent of the user's skill, as well as shorten turnaround time and eliminate several sample handling steps [5]. An important criterion is ease of use, since it has been demonstrated that medical office personnel can produce reliable results when using systems with simple protocols [6]. Test systems using whole blood and requiring no pipetting or diluting steps are also more accurate than systems which require specimen manipulation steps [1].

This paper reports on a near-patient, portable, clinical chemistry system that provides the clinician with rapid availability of *in vitro* diagnostic results. The Piccolo system reports results for panels of blood tests within 14 min of sample application to the consumable. The system consists of the single-use reagent rotor and the Piccolo analyser. An operator applies a few drops of capillary or venous whole blood to the reagent rotor and places it into the analyser. Using capillary action and centrifugal force acting from the centre of the rotor, the system completes all sample processing and optically analyses the cuvettes for multiple chemistries simultaneously. The panel of results is printed on an adhesive-backed card for easy application to patient charts. The design of this system, with emphasis on the consumable, which converts the unmetered blood sample into multiple aliquots of precisely diluted plasma, is described here.

### Materials and methods

*The reagent rotor*

The reagent rotor is an 8 cm diameter consumable containing all the required diluent and dry reagents to perform a panel of tests. Three injection-molded plastic parts are ultrasonically welded together to form the

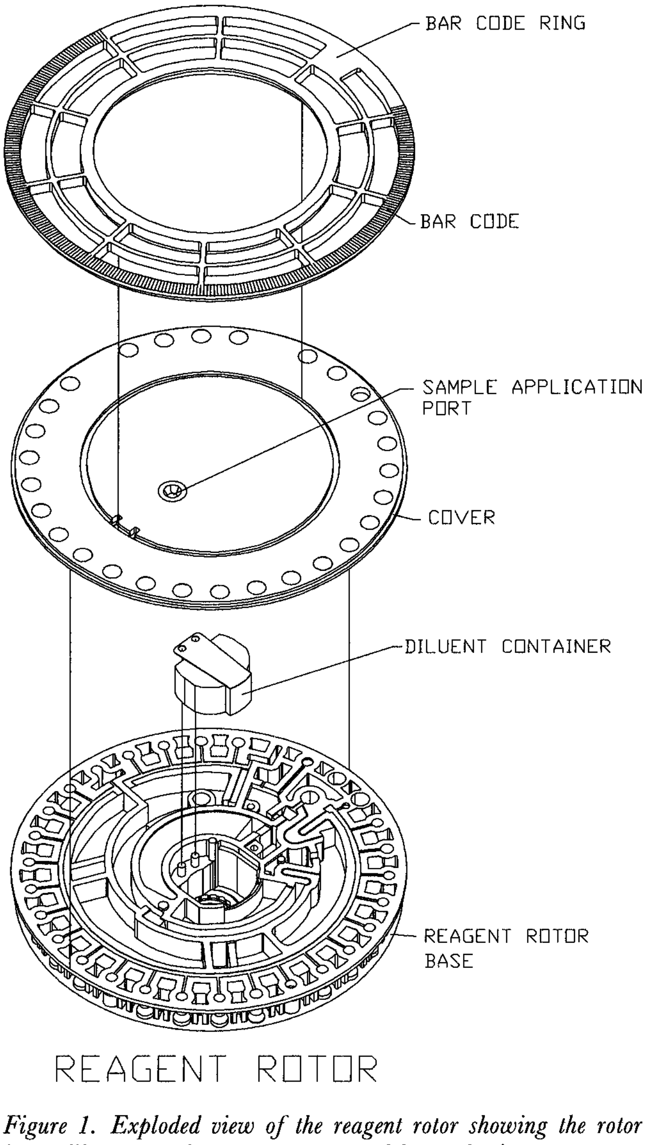

*Figure 1. Exploded view of the reagent rotor showing the rotor base, diluent container, rotor cover, and bar code ring.*

0142-0453/95 $10.00 © 1995 Taylor & Francis Ltd.

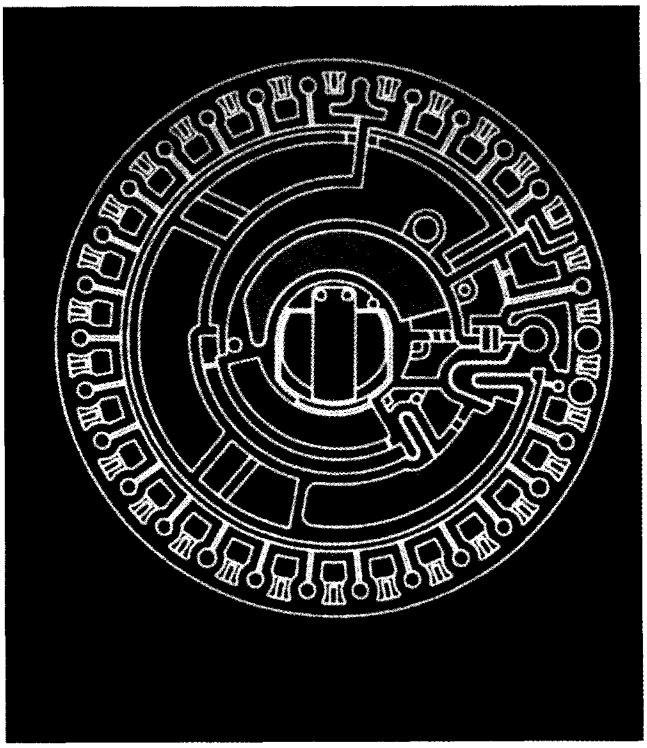

*Figure 2. Schematic diagram of the rotor showing the sample in the application chamber.*

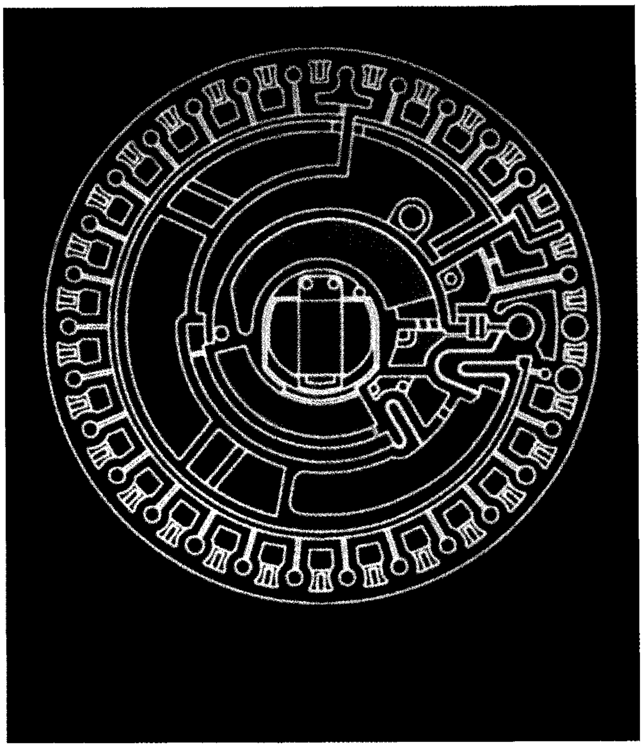

*Figure 4. Schematic diagram of the rotor showing blood entering the plasma metering chamber and diluent being released from the*

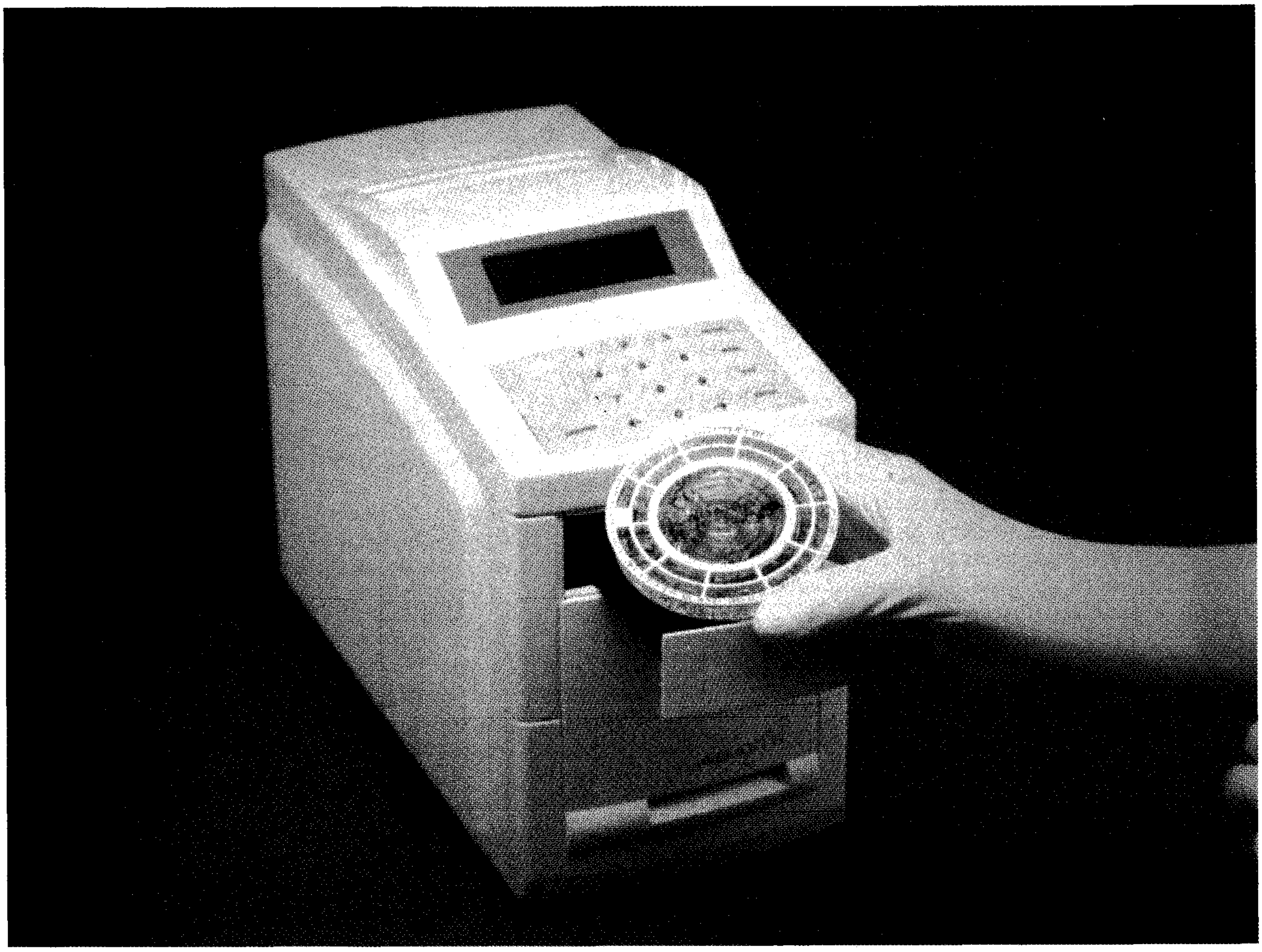

*Figure 3. Placing the rotor into the instrument.*

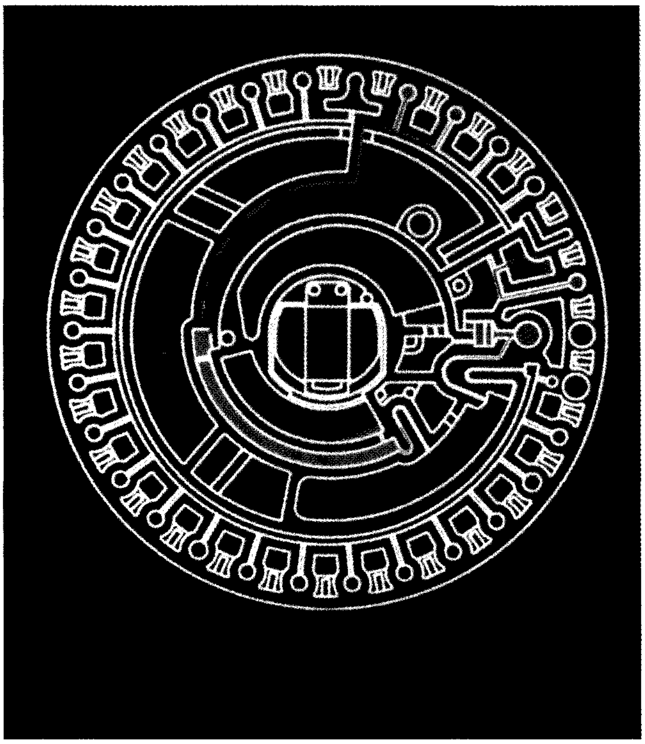

*Figure 5. Schematic diagram of the rotor showing the plasma metering chamber filled and the diluent metered and the system cuvettes filling.*

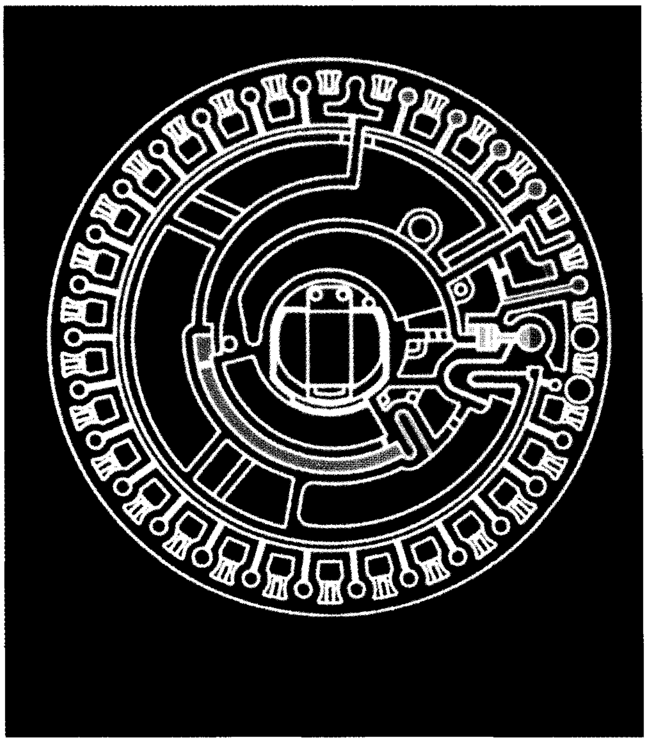

*Figure 6. Schematic diagram of the rotor showing the separation completed and two siphons primed.*

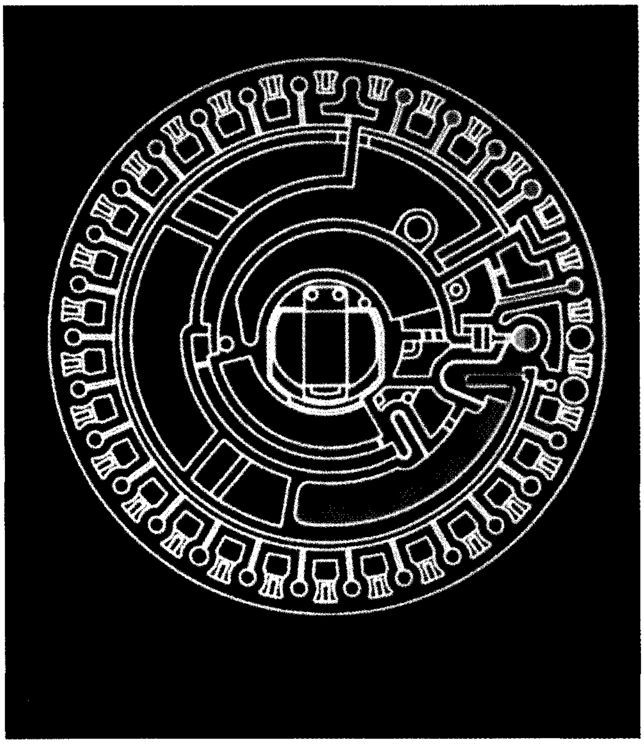

*Figure 7. Schematic diagram of the rotor showing the mixing of the plasma and diluent.*

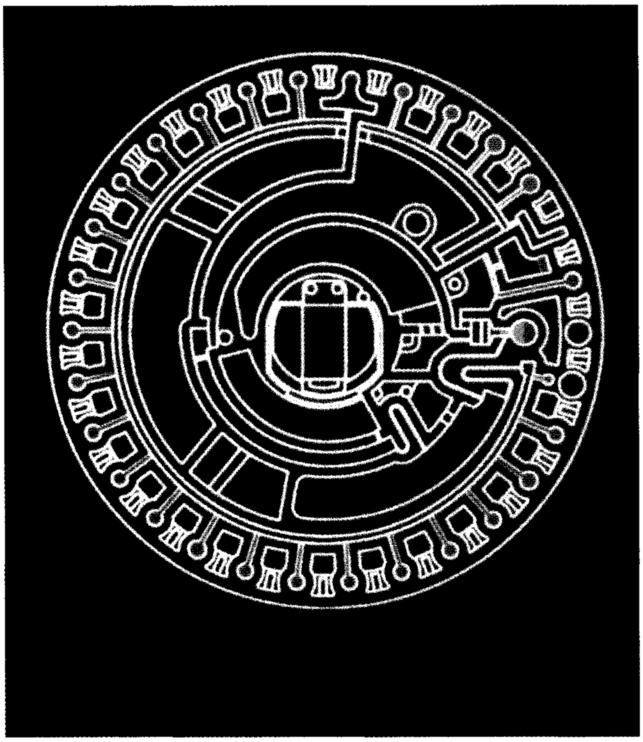

*Figure 8. Schematic diagram of the rotor showing the distribution of diluted plasma to the cuvettes containing reagents.*

reagent rotor. The base and middle layer are molded from polymethylmethacrylate plastic and the top layer is molded from ABS (acrylonitrile, butadiene and styrene) plastic. The welded base and middle layers form the cuvettes, chambers and passageways which allow the fluids to be processed. The top layer protects the cuvette windows from fingerprints, prevents contamination of the analyser by any sample spilled on the rotor surface, and provides imprinted bar-coded, rotor-specific calibration information to the analyser. A sealed container in the centre of the rotor contains 475 μl of diluent. Fluid loss from this container is less than 5 μl per year when stored at 8°C (see figure 1).

The rotor contains 21 cuvettes that will be filled with diluted plasma and four cuvettes that will be filled with diluent. The cuvettes have five pathlengths (1·7 mm, 2·1 mm, 3·1 mm, 4·3 mm and 5·0 mm) to accommodate different reagent sensitivities and analyte concentrations. Premeasured, lyophilized reagent beads for each chemistry in the panel are placed in the cuvettes at the time of manufacture.

### *The analyser*

The analyser is a portable spectrophotometer measuring 24 cm high by 15 cm wide by 30 cm deep; it weighs 6·4 kg. A rotor drawer extending from the front of the analyser positions the rotor on the centre of the spindle at the beginning of each run. The analyser's motor and controller drive the rotor through the profile of rotational speeds needed to process the sample.

The optical system consists of a xenon arc stroboscopic lamp and a beam-splitter/detector capable of reading nine wavelengths. A 16-bit analogue-to-digital converter is used in signal processing. Two microprocessors control the functions of the analyser. A heater maintains the rotor at 37 ± 1°C during the reaction portion of the analysis.

### *The process*

To perform an analysis, the operator obtains a whole blood sample from either a fingerstick or venipuncture. Whole blood samples obtained by venipuncuture can be analysed within 60 min of collection and fingerstick samples should be analysed immediately. A few drops of sample are dispensed, via a microcapillary or other transfer device, into the rotor sample port. Capillary action draws the sample into the rotor. Sufficient sample is applied to the rotor when the leading meniscus of the sample creates a line between two arrows printed on the rotor. The operator need not meter the sample prior to application. A minimum of 90 μl of sample is required and up to 120 μl may be applied to the rotor (see figure 2).

The operator places the rotor with applied sample in the analyser drawer. The drawer closes and the analyser automatically centres the rotor on the spindle. The operator inputs a patient identification number, starts the analysis, and places a results card in the printer. The rotor is now completely controlled by the analyser and no further involvement from the operator is required (see figure 3).

Transparent to the user, the diluent container is opened as the rotor is loaded onto the spindle. The diluent container, a molded high-density polyethylene plastic container, is sealed with polyethylene-laminated aluminum foil. A tab on the foil is folded back across the top of the diluent container and heat staked to the rotor base. A central post on the spindle enters the rotor and pushes the diluent container up. This causes the foil to peel back from the edge and create an opening to release the diluent.

The sample and diluent begin the processing on separate, but parallel, pathways. At the start of analysis, the analyser accelerates the rotor to 5000 r.p.m. counterclockwise and holds this speed for 2·5 min. Centrifugal force causes the diluent to be thrown from the diluent container into a holding chamber. The small exit channel at the most radially outward point of the chamber allows the diluent to fill a metering chamber in a controlled manner. The chamber is filled completely with 356·25 μl of diluent. The remaining diluent overflows this chamber and moves through a channel which sequentially fills four cuvettes and places the remaining diluent into a dump isolated from the rest of the rotor. The four cuvettes are used for internal rotor quality control.

Simultaneously with diluent metering, centrifugal forces causes the sample to exit the application chamber and move through a small channel into the plasma metering chamber. The chamber is filled completely by 75 μl of sample and the remainder overflows into a 'sufficient sample' cuvette. Any excess sample is trapped in an isolation dump. If no fluid is detected in the 'sufficient sample' cuvette, the analysis is aborted due to a deficient quantity of sample. The precise quantity of blood metered into the plasma metering chamber is separated by centrifugal force into plasma and red blood cells. Adequate separation is achieved in 30 s for most samples. Samples with higher haematocrits require longer separation and packing times to provide the necessary 20 μl of clear plasma. Spinning the rotor for 2·5 min is sufficient to separate samples up to a haematocrit of 62%. (See figures 4 and 5.)

The next step combines the diluent and plasma. A siphon entrance of capillary dimension, is located at the radially outermost point of the diluent metering chamber. Another siphon entrance is located partway down the plasma metering chamber. Neither of these siphons fills during the first spin because the centrifugal force is far higher than the capillary force. After the separation step is completed, the rotor stops spinning and capillary forces pull the fluids over the bend in the siphons. Both siphons exit into a single mixing chamber (see figure 6).

The rotor is now spun at 5000 r.p.m. clockwise. The diluent metering chamber is completely emptied since the exit of the siphon is further from the center of the rotor than the extreme edge of the diluent metering chamber. The plasma metering chamber is emptied to a radial distance equaling the placement of the exit of the plasma metering siphon. The volume of plasma metered is 18·75 μl. The remaining 56·25 μl of sample and red blood cells is trapped in the lower portion of the plasma metering chamber.

The rotor speed is varied to mix the metered diluent and plasma in the mixing chamber. The rotor abruptly brakes to a speed of 750 r.p.m. and then slowly climbs back to

4000 r.p.m., this pattern is repeated for 15 cycles. The abrupt deceleration provides sufficient tangential force to move and mix the fluid in the mixing chamber. A minimum speed of 750 r.p.m. is required during the mixing process, to ensure that the applied centrifugal force exceeds the capillary strength of final siphon. This mixing profile prevents priming of the siphon until the fluids are homogeneously mixed (see figure 7).

The final siphon is primed by capillary action after mixing is complete and the rotor is stopped. The rotor is then spun at 3000 r.p.m. clockwise for 40 s. The diluted plasma flows out of the mixing chamber and into a distribution channel which leads to 21 cuvettes and an isolation dump. The 21 cuvettes are filled sequentially and the remaining diluted plasma flows into the dump (see figure 8).

Each cuvette has a single channel for both flow of fluid into the cuvette and air venting. Under highly controlled conditions, the fluid flows down one side of the inlet channel while air vents from the other side. No special features of the cuvette inlet channel are required to achieve this control. The size of the fluid stream is controlled by the resistance of the siphon, the rotational speed of the rotor, and the pressure head of the remaining fluid. The rotor must also spin fast enough to overcome the capillary strength of the inlet channel which is 0·50 mm wide and 0·13 mm high. Each cuvette contains one or two beads of lyophilized reagents appropriate for the particular test to be run within that cuvette; these beads dissolve completely in the time required to fill the cuvette.

After all of the cuvettes are filled, and the excess diluted plasma is isolated in the dump, the rotor is oscillated from 1000 r.p.m. clockwise to 1000 r.p.m. counterclockwise for 70 s. This oscillation cycle creates swirl patterns in the cuvettes which mix the chemistry and the diluted plasma.

The spectrophotometer monitors the reactions in all of the cuvettes for 3·5 min by flashing the xenon arc lamp synchronously with the spinning rotor. The lamp is flashed approximately 5000 times for each rotor.

The analyser detects the presence of each cuvette by sensing 45° wedges of plastic placed every 12° around the periphery of the rotor. On each spin, the processor selects which cuvette to flash and which of the nine wavelengths to measure.

The results are calculated and printed on an adhesive-backed card for easy attachment to the patient's files (see figure 9). In addition, the results can be uploaded to a computer via an RS-232 port.

### Results

### *The rotor*

The rotor's contribution to total system imprecision is in the reproducibility of cuvette pathlength between rotors; and the precision of metering plasma and diluent and homogeneously mixing the two fluids.

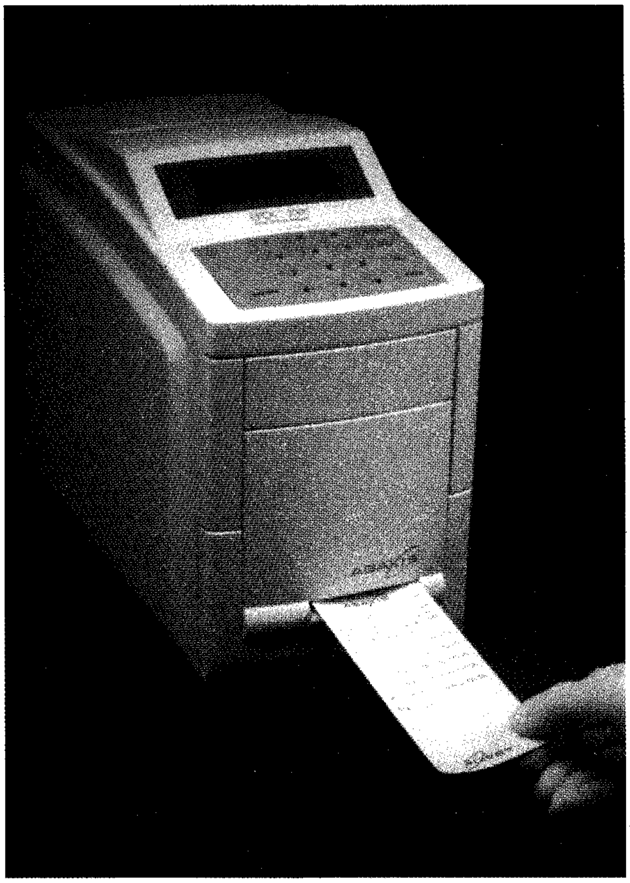

*Figure 9. Results card is printed.*

### *Reproducibility of cuvette pathlength*

The pathlength of all 25 cuvettes was measured on 82 ultrasonically welded rotors. The 82 rotors include examples welded on two different welders, on different days and from different lots of molded rotors. The coefficient of variation (CV) of the most precise cuvette was 0·07% and the least precise cuvette was 0·47%.

### *Precision of fluid metering and mixing*

The goal for precision of metering plasma and diluent and mixing them homogeneously was 2% or better. The precision of the entire system was measured to obtain an indication of the precision of the rotor's fluid functions, since the contribution of error from the pathlength and the analyser was minimal. Within-day system precision was obtained using the glucose method and plasma. Four rotors were run on eight analysers for a total sample size of 32. The mean was 115 mg/dl (6·4 mmol/l) and the CV was 1·0%. Plasma was used as the sample to ensure that the glucose value would be stable for the duration of the study.

The contributions to error in this measurement include: plasma metering; diluent metering; mixing homogeneity; pathlength of the reagent and sample blank cuvettes; reagent imprecision; instrument-to-instrument variation; instrument noise; and changes within the controls run on the analyser.

*Table 1. Day-to-day reproducibility investigation.*

| Chemistry | Replicates | Mean | CV |
|---|---|---|---|
| Glucose—normal | 120 | 70 mg/dl (3·9 mmol/l) | 1·0% |
| Glucose—abnormal | 120 | 260 mg/dl (14·4 mmol/l) | 1·6% |
| AST—normal | 120 | 40 U/l | 3·2% |
| AST—abnormal | 120 | 150 U/l | 1·7% |
| Uric acid—normal | 120 | 3·7 mg/dl (0·22 mmol/l) | 6·3% |
| Uric acid—abnormal | 120 | 7·6 mg/dl (0·46 mmol/l) | 3·1% |
| Total bilirubin—normal | 120 | 1·0 mg/dl (17·1 μmol/l) | 7·5% |
| Total bilirubin—abnormal | 120 | 5·6 mg/dl (95·8 μmol/l) | 5·0% |
| Total protein—normal | 120 | 6·6 g/dl (66 g/l) | 1·3% |
| Total protein—abnormal | 120 | 4·6 g/dl (46 g/l) | 2·3% |

*Table 2. Total imprecision.*

| Chemistry | Replicates | Mean | CV |
|---|---|---|---|
| Glucose—normal | 80 | 75 mg/dl (4·2 mmol/l) | 1·9% |
| Glucose—abnormal | 80 | 261 mg/dl (14·5 mmol/l) | 1·2% |
| AST—normal | 80 | 47 U/l | 3·9% |
| AST—abnormal | 80 | 145 U/l | 3·2% |
| Uric acid—normal | 80 | 3·8 mg/dl (0·23 mmol/l) | 4·8% |
| Uric acid—abnormal | 80 | 7·5 mg/dl (0·45 mmol/l) | 3·9% |
| Total bilirubin—normal | 80 | 1·3 mg/dl (22·2 μmol/l) | 5·1% |
| Total bilirubin—abnormal | 80 | 5·5 mg/dl (94·0 μmol/l) | 5·1% |
| Total protein—normal | 80 | 6·2 g/dl (62 g/l) | 2·0% |
| Total protein—abnormal | 80 | 4·4 g/dl (44 g/l) | 2·0% |

### *The system*

The reproducibility of the system for a panel of five tests was measured using Dade Monitrol ES Level I and Level II Chemistry Controls. A 15-day precision study was conducted using 12 different Piccolo analysers. A total of 120 rotors were run using each control. The day-to-day reproducibility is shown in table 1.

A 20-day precision study was conducted by the Scripps Clinic (La Jolla, CA, USA) using a single Piccolo analyser. The study was conducted in accordance with the guidelines published by the National Committee for Clinical Laboratory Standards. The total imprecision for each method in the rotor is shown in table 2.

### Discussion

The precision of metering and mixing plasma and diluent is better than the 2% CV.

It has been demonstrated that a panel of clinical chemistry results can be assayed from whole blood using capillary action and centrifugal force acting from the centre of the reagent rotor. This meant that a compact analyser resulting in a portable, convenient system could be developed. The menu of tests is being expanded and several different panels will be available.

### Acknowledgements

The authors wish to acknowledge the efforts of many people who contributed to the development of this system; particularly, Apex Machine Tool Co. (Farmington, CT, USA) and Nypro, Inc. (Clinton, MA, USA) for their expertise in toolmaking and injection molding respectively. They also thank Ms L. Swanson for her help in editing this manuscript.

### References

1. Zaloga, G. P., *Chest*, **97** (1990), 185S.
2. Baer, D. M. and Belsey, R. E., *Chest*, **97** (1990), 191S.
3. Misiano, D. R., Meyerhoff, M. E. and Collison, M. E., *Chest*, **97** (1990), 204S.
4. Salem, M., Chernow, B., Burke, R., Stacey, J., Slogoff, M. and Sood, S., *Journal of the American Medical Association*, **266** (1991), 382.
5. Broughton, P. M. and Buckley, B. M., *Scandinavian Journal of Clinical Laboratory Investigation*, **47** (1982), 99.
6. Nanji, A. A., Poon, R. and Hinberg, I., *Canadian Medical Association Journal*, **138** (1988), 517.

